# Media Use during Childhood and Adolescence

**DOI:** 10.3390/ijerph18030967

**Published:** 2021-01-22

**Authors:** Elena Bozzola

**Affiliations:** Pediatric Diseases Unit, Bambino Gesù Children’s Hospital, IRCCS, Piazza Sant Onofrio 4, 00165 Roma, Italy; elena.bozzola@opbg.net; Tel.: +39-0668592744

Media devices use among children and adolescents is rapidly increasing due to the small size, which allows mobility, interactivity, and easiness to benefit from free content and applications. In recent years, the decreasing costs contributed to the easy access to technology by families. Children use mobile devices to play games or to watch videos, to take pictures and record video, to access content and applications. Nevertheless, with the rise of social gaming, the line between entertainment and socializing has become blurred. The Internet is entertaining and helping children and adolescents to release emotions and enhance self-esteem. Nevertheless, excessive users will be more focused on the Internet and less interested in real life, escaping in a digital world. With the growing increase in screen time of kids and, therefore, the high chances of them being more vulnerable to online risks, supervision is mandatory. Families need to pay attention, not only to the time their kids spend on media devices, but also on the content, because children and adolescents are in the stage of psychological development. The Italian Pediatric Society warn families on the negative consequences related to incorrect use of media devices and on its abuse. In fact, mainly in early childhood, media device use may lead to learning difficulties, and interfere with brain development and relationships with parents and peers. Scientific studies demonstrated that media use by children and toddlers could have positive effects only with both adequate content and parental interaction, which contributes to behavioral and neurocognitive system development [1]. As for adolescence, social media technology and problematic smartphone use may lead to learning consequences, such as low academic outcomes, reduced concentration, procrastination and even cyberbullying [2,3]. Balancing benefits and harms, social media, such as Instagram, may represent a great chance for self-expression and change in adolescents, but the strenuous search for confirmation may increase the risk of social isolation and a decrease in self-acceptance [4].

The COVID-19 pandemic has had a dramatic effect on Internet usage, as the online traffic has rapidly increased. In the lockdown period, due to COVID-19 infection, digital devices allowed children and adolescents to connect with others on an emotional level, even if this did not replace human contact. Social network use helped in maintaining relationships with grandparents, sick relatives or peers in quarantine. Keeping in touch with them helped in reducing loneliness despite physical distance. Moreover, digital platform use substituted frontal lessons during school closure, avoiding educational disrupting.

Studies revealed that adolescents cleverly used the Internet, demonstrating interest in health and willingness to engage in medicine in the future [5].

However, school closure and self-isolation for a long time may influence adolescent behaviour, and mental and emotional well-being. In fact, lockdown may restrict their movements to a limited space and a monotonous daily routine. In this context, the long period of separation from the outside world may negatively impact on children and adolescent’s mental health status, experiencing fear, loneliness, panic, and anxiety amid the COVID-19 outbreak. In most cases, children and adolescents used the Internet massively during the pandemic, and the hours of use of recreational electronic devices, mainly in the late evening, were longer than before, with an increased risk of negative consequences. Media usage may interfere with sleep quality through the increase in psychophysiological arousal, caused by either the content or a prolonged bright light exposure, and through reduced sleep quality or nightmares [1]. As the circadian rhythm is negatively influenced by pre-sleep smartphone use, adolescents may experience a high risk of sleeping problems, such as increased sleep latency, arousal and reduced sleep duration [2]. During the outbreak of the COVID-19 pandemic, adolescents experienced disrupted sleep patterns and a poor sleeping quality with irregular wake up time [6]. During the COVID-19 outbreak, studies reported 6.2% addicted Internet users and 46.0% problematic Internet users [7]. Social isolation due to the COVID-19 pandemic and the intensive use of the Internet among children and adolescents may have negative consequences for the practice of self-inflicted violence. An incorrect use of media devices may also contribute to depression, which has been reported in over 40% of adolescents during the COVID-19 outbreak. Female sex, adolescence (age 15–18 years), distant learning, a daily sleep duraotion less than 6 h, and physical exercise less than 30 min a day have been indicated as risk factors for depression [8].

On the other hand, light-to-moderate intensive physical exercise three times a week proved to be a promising antidepressant treatment for adolescents in 6–12 weeks.

In conclusion, even if media devices and digital communication may be useful tools to communicate and to avoid barriers during public emergencies, warning families of the hidden risk of incorrect use is mandatory by health care providers.

## Data Availability

At Bozzola’s office.
